# Association between remnant cholesterol levels and reversion to normoglycemia from prediabetes: a 5-year longitudinal cohort study of Chinese non-obese adults

**DOI:** 10.3389/fendo.2025.1510470

**Published:** 2025-07-30

**Authors:** Wei Liu, Wenjing Jian, Suina Lin, Zhenhua Huang

**Affiliations:** ^1^ Department of Emergency Medicine, Huangpu People’s Hospital of Zhongshan, Zhongshan, China; ^2^ Department of Integrated Traditional Chinese and Western Medicine, The First Affiliated Hospital of Shenzhen University &Shenzhen Second People’s Hospital, Shenzhen, China; ^3^ Department of Ophthalmology, Huangpu People’s Hospital of Zhongshan, Zhongshan, China; ^4^ Department of Emergency Medicine, Pengpai Memorial Hospital, Shanwei, China; ^5^ Department of Emergency Medicine, The First Affiliated Hospital of Shenzhen University &Shenzhen Second People’s Hospital, Shenzhen, China

**Keywords:** remnant cholesterol, reversion, normoglycemia, prediabetes, non-obese adults

## Abstract

**Purpose:**

The primary objective of this study is to explore the relationship between remnant cholesterol (RC) levels and the reversion to normoglycemia in non-obese Chinese individuals with prediabetes.

**Methods:**

To achieve this goal, we conducted a retrospective cohort study involving 8,109 non-obese prediabetic participants in China, using the Cox proportional hazards regression model to analyze the correlation between RC and the likelihood of returning to normoglycemia.

**Results:**

The results indicate a significant negative correlation between RC levels and reversion to normoglycemia (HR=0.49, 95% CI: 0.47-0.52). Specifically, as RC quartiles increase, the probability of reverting to normoglycemia significantly decreases, with participants in the highest quartile having a 51% lower likelihood of recovery compared to those in the lowest quartile. Furthermore, we identified a nonlinear relationship between RC and the reversion to normoglycemia, with 1.10 mmol/L established as the inflection point. When RC levels are below this threshold, decreasing RC significantly increases the likelihood of recovery. To further validate the robustness of our findings, we conducted sensitivity and subgroup analyses, all of which support the reliability of the main results.

**Conclusion:**

There exists a significant negative and nonlinear relationship between RC levels and the reversion to normoglycemia in non-obese Chinese prediabetic patients. This suggests that lowering RC levels may play an important role in reversion to normoglycemia from prediabetes.

## 1 Introduction

Diabetes mellitus (DM) has become a global public-health crisis. The International Diabetes Federation (IDF) projects that the number of people living with type 2 diabetes will surge over the next two decades, rising from 425 million in 2017 to 629 million by 2045 (1). This epidemic continues to afflict adults and is now spreading at an alarming rate to adolescents (2), with obesity widely regarded as the principal driver (3, 4). Yet it must not be overlooked that 10–20% of patients are non-obese, and in some parts of Asia this group even constitutes many cases (5). Prediabetes is characterized by elevated blood sugar levels that are not sufficiently high to be diagnosed as diabetes. It is linked to a higher risk of diabetes. The World Health Organization (WHO) defines prediabetes as impaired glucose tolerance (IGT) or impaired fasting glucose (IFG) (6). In 2017, IDF estimated that approximately 374 million adults worldwide had prediabetes, representing 7.7% of the adult population. By 2045, prediabetes cases are projected to reach 548 million, representing 8.4% of the global adult population (7). Prediabetes poses a significant risk, with an annual 5%-10% chance of progressing to diabetes and up to 70% of individuals eventually developing the disease (8). However, a proportion of individuals with prediabetes can delay or even reverse progression to diabetes through dietary changes, increased physical activity, and other lifestyle interventions (9, 10). Thus, reversing prediabetes and restoring normal blood sugar levels are crucial for preventing diabetes and its complications.

Residual cholesterol (RC) has been highly emphasized due to the residual risks it causes, for example heart disease, stroke, and diabetes (11–14). Research indicates that RC has a stronger positive correlation with diabetes, prediabetes, and IR compared to traditional lipids and lipid ratios in the general population (15). And in Chinese adults with prediabetes, higher residual cholesterol levels are associated with a lower probability of normalizing blood glucose (16). Unfortunately, there is not clear in the non-obese individuals. This cohort study aims to investigate the association between RC and the likelihood of non-obese individuals with prediabetes reverting to normoglycemia, using existing data.

## 2 Methods

### 2.1 Study design and data source

This study employs a retrospective cohort approach, leveraging a substantial dataset from a large-scale study conducted by the Rich Healthcare Group in China (17). This foundation ensures the robustness of our research. The dataset used in this study was provided by Chen et al. (17) and is accessible through the DATADRYAD repository, thereby ensuring data verifiability and transparency.

### 2.2 Study population

**Figure 1:**
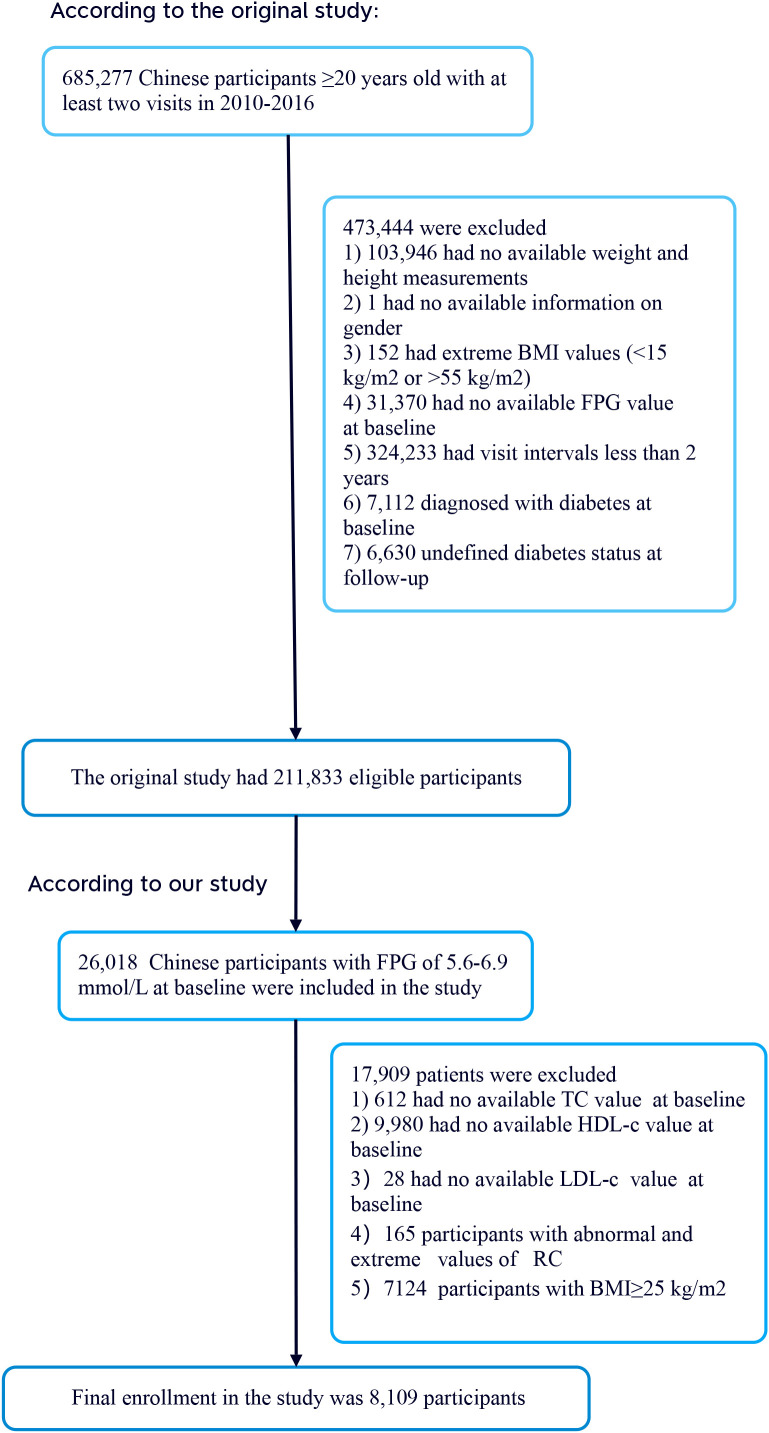
Overview of the process used for screening study participants.

The researchers extracted data from a computer database established by the Rich Healthcare Group in China, which contains all medical records of participants who underwent health checks from 2010 to 2016, covering 32 regions and 11 cities in China (17). Additionally, the initial study included 26,018 participants with baseline fasting plasma glucose (FPG) levels between 5.6–6.9 mmol/L. The initial study cohort comprised 685,277 participants, all aged 20 and above, who had at least two visits between 2010 and 2016. During the study, 473,444 participants were excluded, leaving 211,833 individuals for analysis. The exclusion criteria included 103,946 participants with missing weight and height measurements; one participant with missing gender information; 152 participants with extreme BMI values (<15 kg/m² or >55 kg/m²); 31,370 participants lacking baseline FPG values; 324,233 participants with less than a 2-year interval between visits; 7,112 participants who were diagnosed with diabetes at baseline; and 6,630 participants with an unclear diabetes status at follow-up. Additionally, the initial study included 26,018 participants with baseline FPG levels between 5.6–6.9 mmol/L (18). According to the 2021 standards of the American Diabetes Association, this range is defined as prediabetes. Subsequently, 612 participants with missing total cholesterol (TC) values, 9,980 participants with missing high-density lipoprotein cholesterol (HDL-C) values, 28 participants with missing low-density lipoprotein cholesterol (LDL-C) values, 165 participants with abnormal or extreme RC values, and 7,124 participants with BMI ≥ 25 kg/m² were excluded. Ultimately, 8,109 participants were included in the secondary analysis. **Figure 1** details the entire process of participant selection.

### 2.3 Ethics statement

The audit board of the Rich Healthcare Group approved the original study, and data collection was conducted retrospectively. Furthermore, the institutional ethics committee waived the requirement for study approval and informed consent for this retrospective study (17). The study strictly adhered to the principles outlined in the Declaration of Helsinki, and all methods employed were in accordance with relevant guidelines and regulations, with detailed specifications provided in the declaration section. Additionally, the secondary analysis complied with these ethical standards.

### 2.4 Data collection

In this study, trained investigators used standardized questionnaires to collect comprehensive baseline data from participants. This data included demographic characteristics such as age and gender, as well as lifestyle factors such as smoking and drinking habits, and family history information related to diabetes. For BMI, the study calculated it as the weight (in kilograms) divided by the square of the height (in meters). Simultaneously, blood pressure was measured precisely using a standard mercury sphygmomanometer. Following each visit, the research team collected venous blood samples from participants after at least 10 hours of fasting. Subsequently, these blood samples were analyzed for FPG, triglycerides (TG), TC, HDL-C, LDL-C, alanine aminotransferase (ALT), blood urea nitrogen (BUN) and creatinine (Scr) using a Beckman 5800 automated analyzer (17).

### 2.5 Variables and outcome measure

RC was recorded as a continuous variable, and its definition process (calculated as RC = TC – HDL – LDL) has been detailed in the relevant literature and applied in this study (19). The primary focus of this study was the restoration of blood glucose levels to normal. The criteria for transitioning from prediabetes to normal blood glucose during follow-up were defined as participants not being diagnosed with diabetes and maintaining FPG levels below 5.6 mmol/L.

### 2.6 Covariates

Covariate selection was informed by clinical expertise and prior research outcomes (20–24). In this study, the chosen covariates were mainly divided into two categories: (1) continuous variables, including age, height, weight, BMI, ALT, systolic blood pressure (SBP), diastolic blood pressure (DBP), BUN, Scr, HDL-C, LDL-C, TG, and TC; and (2) categorical variables, involving gender, family history of diabetes, drinking status, and smoking status. Smoking status was classified into four groups: current, former, non-smoking, and unknown (lacking smoking data). Correspondingly, drinking status was similarly divided into four categories: current, former, non-drinking, and unknown (denoting missing drinking data).

### 2.7 Missing data processing

The study recorded missing data for SBP, DBP, ALT, BUN, and Scr in 1 (0.01%), 1 (0.01%), 23 (0.28%), 212 (2.61%), and 65 (0.80%) participants, respectively. To address the impact of missing data, we utilized multiple imputation techniques. The imputation model accounted for a range of factors, such as gender, age, BMI, blood pressure, lipid profiles, and health behaviors. The model was refined via five iterative regressions, applying the missing at random (MAR) assumption for all analyses.

### 2.8 Statistical analysis

Non-obese prediabetic participants were divided into four quartiles based on their RC (insulin sensitivity) levels, and comparisons were made between variables within these quartiles. Normally distributed continuous variables were reported with means and standard deviations, while skewed variables were described using medians and interquartile ranges. Frequencies and percentages were used to present categorical variables. The Kruskal-Wallis H test was employed for skewed continuous variables, and one-way ANOVA was used for normally distributed ones. Chi-square tests were performed on categorical data. The Cox proportional hazards regression model was used to investigate the association between RC and the likelihood of normalizing blood glucose in non-obese prediabetic individuals, with three adjustment strategies: Model I (no covariates), Model II (gender and age), and Model III (comprehensive covariates including age, gender, SBP, DBP, BMI, ALT, BUN, Scr, TG, family history of diabetes, drinking habit, smoking habit, and FPG). Furthermore, the Cox proportional hazards regression model with cubic splines was utilized to explore the nonlinear relationship between RC and the probability of normalizing blood glucose in non-obese prediabetic participants, identifying potential nonlinear inflection points via recursive algorithms. Multivariate the Cox proportional hazards regression analyses were performed on groups below and above the inflection points to calculate unique risk ratios for each subgroup. Log-likelihood ratio tests identified the most effective model, explaining the relationship between RC and the transition from prediabetes to normal blood glucose in non-obese individuals. Sensitivity and subgroup analyses were conducted, initially focusing on participants with a BMI above 18 kg/m². In sensitivity analysis, participants with a SBP below 140 mmHg were excluded. The study also examined the relationship between RC and the normalization of blood glucose in individuals under 60 years old. General additive modeling (GAM) was used to better integrate various continuous covariates. All analyses were performed using Empower Stats software, with statistical significance assessed at P < 0.05 for two-tailed tests.

## 3 Results

### 3.1 Characteristics of patients with prediabetes

**Table 1** presents the demographic and clinical profiles of the study participants. The study enrolled 8,109 participants with an average age of 50.29 years and a standard deviation of 13.85 years. Among non-obese participants, the median residual cholesterol (RC) was 0.63, and the interquartile range exhibited variability. The median follow-up duration of the study was 2.87 years, with 3,976 participants (48.99%) ultimately normalizing their blood glucose levels. Participants were categorized into four quartiles based on their residual cholesterol (RC) levels: Q1 (<0.38), Q2 (0.38-0.62), Q3 (0.62-0.91), and Q4 (≥0.91). Compared to the lowest quartile (Q1 <0.38), participants in the highest quartile (Q4 ≥0.91) had significantly higher baseline measurements of age, height, weight, BMI, SBP, DBP, TC, TG, LDL-C, aminotransferase levels (including ALT, Scr and FPG. Conversely, participants in the highest quartile exhibited a trend towards lower high-density lipoprotein HDL-C levels. Additionally, the proportion of males and current smokers was higher in the Q4 group. Notably, the distribution of RC was skewed, ranging from 0.01 to 6.85 mmol/L, with a median of 0.63 mmol/L (see **Supplementary Figure S1**).

**Table 1:** The baseline characteristics of participants.

RC (quartile) (mmol/L)	Q1 (<0.38)	Q2 (0.38-0.62)	Q3 (0.62-0.91)	Q4 (≥0.91)	P-value
Participants	1,961	2,090	1,996	2,062	
Age (years)	47.16 ± 13.91	49.35 ± 13.94	51.29 ± 13.61	53.26 ± 13.20	<0.001
Height (cm)	165.38 ± 8.20	165.49 ± 8.38	166.16 ± 8.33	166.49 ± 8.56	<0.001
Weight (kg)	59.88 ± 8.31	61.37 ± 8.38	62.56 ± 8.10	63.72 ± 8.28	<0.001
BMI (kg/m2)	21.82 ± 2.00	22.32 ± 1.84	22.58 ± 1.69	22.91 ± 1.61	<0.001
SBP (mmHg)	122.16 ± 17.24	124.01 ± 17.64	125.17 ± 17.39	126.48 ± 17.78	<0.001
DBP (mmHg)	74.56 ± 10.44	75.95 ± 10.59	76.62 ± 10.40	77.47 ± 10.95	<0.001
TC (mmol/L)	4.45 ± 0.78	4.77 ± 0.80	5.07 ± 0.81	5.63 ± 0.98	<0.001
TG (mmol/L)	0.96 ± 0.48	1.21 ± 0.66	1.53 ± 0.77	2.41 ± 1.73	<0.001
HDL-C (mmol/L)	1.50 ± 0.30	1.43 ± 0.27	1.34 ± 0.28	1.26 ± 0.28	<0.001
LDL-C (mmol/L)	2.72 ± 0.64	2.84 ± 0.67	2.96 ± 0.69	3.06 ± 0.83	<0.001
RC (mmol/L)	0.24 ± 0.10	0.51 ± 0.07	0.76 ± 0.08	1.31 ± 0.45	<0.001
ALT (U/L)	16.30 (12.10-22.10)	17.15 (13.00-24.00)	19.00 (14.00-26.00)	21.35 (16.00-30.70)	<0.001
BUN (mmol/L)	4.83 ± 1.18	4.91 ± 1.25	4.96 ± 1.24	4.96 ± 1.27	0.002
Scr (μmol/L)	69.42 ± 15.73	70.59 ± 15.46	71.31 ± 16.68	72.03 ± 16.06	<0.001
FPG at baseline (mmol/l)	5.90 ± 0.29	5.91 ± 0.30	5.92 ± 0.30	5.97 ± 0.33	<0.001
Gender (n, %)					<0.001
Male	962 (49.06%)	1145 (54.78%)	1175 (58.87%)	1277 (61.93%)	<0.001
Female	999 (50.94%)	945 (45.22%)	821 (41.13%)	785 (38.07%)	
Smoking status (n, %)					<0.001
Current smoker	77 (3.93%)	102 (4.88%)	138 (6.91%)	178 (8.63%)	
Ever smoker	26 (1.33%)	23 (1.10%)	31 (1.55%)	22 (1.07%)	
Never	416 (21.21%)	493 (23.59%)	421 (21.09%)	489 (23.71%)	
Unknown	1442 (73.53%)	1472 (70.43%)	1406 (70.44%)	1373 (66.59%)	
Drinking status (n, %)					<0.001
Current drinker	6 (0.31%)	20 (0.96%)	20 (1.00%)	28 (1.36%)	
Ever drinker	85 (4.33%)	101 (4.83%)	111 (5.56%)	118 (5.72%)	
Never	428 (21.83%)	497 (23.78%)	459 (23.00%)	543 (26.33%)	
Unknown	1442 (73.53%)	1472 (70.43%)	1406 (70.44%)	1373 (66.59%)	
Family history of diabetes (n, %)					0.898
No	1911 (97.45%)	2030 (97.13%)	1945 (97.44%)	2005 (97.24%)	
Yes	50 (2.55%)	60 (2.87%)	51 (2.56%)	57 (2.76%)	
Year of follow-up	2.67 ± 0.77	2.83 ± 0.84	2.99 ± 0.91	3.24 ± 1.04	<0.001

Continuous variables were summarized as mean (SD) or medians (quartile interval); categorical variables were displayed as percentage (%). BMI, body mass index; SBP, systolic blood pressure; DBP; diastolic blood pressure; TC, total cholesterol; TG triglyceride; HDL-C, high-density lipoprotein cholesterol; LDL-C, low-density lipoprotein cholesterol; ALT, alanine aminotransferase; BUN, blood urea nitrogen; Scr, serum creatinine; FPG, fasting Plasma glucose; RC, remnant cholesterol.

### 3.2 The rate of return to normoglycemia from prediabetes in non-obese individuals

During a median follow-up of 2.87 years, 3,973 participants with prediabetes achieved normal blood glucose levels, corresponding to a recovery rate of 166.92 per 1,000 person-years. The normalization rates for blood glucose varied across RC quartiles: Q1, 210.86; Q2, 181.24; Q3, 154.31; Q4, 131.11. Overall, 48.99% of participants with prediabetes transitioned to normal blood glucose. The transition rates to normal blood glucose were 56.30% for Q1, 51.29% for Q2, 46.14% for Q3, and 42.48% for Q4. Participants in the highest RC quartile (Q4) exhibited a significantly lower transition rate to normal blood glucose compared to those in the lowest quartile (Q1) (p < 0.001), as detailed in **Table 2** and **Supplementary Figures 2, 3** presents the Kaplan-Meier survival curves illustrating the probability of prediabetic non-obese participants transitioning to normal blood glucose, stratified by RC quartiles. The log-rank test revealed significant differences in the probability of normalizing blood glucose across RC quartiles (P < 0.001). Throughout follow-up, participants in RC quartiles Q2 to Q4 had significantly reduced odds of recovering to normal blood glucose compared to those in Q1.

**Table 2:** Rate of return to normoglycemia from prediabetes (% or per 1000 person-years).

RC (quartile)	Participants (n)	Reversion events (n)	Reversal rate (95%CI) (%)	Per 1000 person-year
Total	8,109	3,973	48.99 (47.91-50.08)	166.92
Q1	1,961	1,104	56.30 (54.10-58.49)	210.86
Q2	2,090	1072	51.29 (42.05-45.21)	181.24
Q3	1,996	921	46.14 (43.96-48.33)	154.31
Q4	2,062	876	42.48 (40.35-44.62)	131.11
P for trend			<0.001	

### 3.3 Factors influencing the regression from prediabetes to normoglycemia were analyzed using univariate the Cox proportional hazards regression

the Cox proportional hazards regression was used to examine factors affecting the transition from prediabetes to normal blood glucose levels. The univariate analysis found no significant associations between smoking status (high, ever smoked, unknown), alcohol consumption, and the recovery from prediabetes to normal blood glucose (all P > 0.05). However, the results showed that age, gender, weight, BMI, SBP, DBP, TC, TG, HDL-C, LDL-C, RC, FPG, ALT, BUN, Scr, never smoking, and a family history of diabetes were positively associated with the transition to normal blood glucose (all P < 0.05; see **Supplementary Table S1**).

### 3.4 Multivariable analyses were conducted using the Cox proportional hazards regression models

We performed a multivariate analysis using the Cox proportional hazards regression to investigate the association between RC and the recovery of normal blood glucose in prediabetic patients (**Table 3**). The unadjusted model showed a significant negative correlation between an increase in RC and the risk of normalizing blood glucose, with an HR of 0.49 (95% CI 0.47–0.51, P < 0.001). This trend was also significant in the minimally adjusted model (Model II), with an HR of 0.56 (95% CI 0.54–0.58, P < 0.001) for each unit increase in RC. In the fully adjusted model (Model III), the association remained, with an HR of 0.49 (95% CI 0.47–0.52, P < 0.001) for each unit increase in RC. Subsequently, RC was converted from a continuous to a categorical variable and included in the Cox proportional hazards regression model. In Model III, the HRs for the risk of normalizing blood glucose across RC quartiles were: Q2, 0.80 (95% CI 0.77, 0.83); Q3, 0.62 (95% CI 0.60, 0.65); and Q4, 0.48 (95% CI 0.46, 0.50). This suggests that the likelihood of normalizing blood glucose decreased by 20% in the second quartile, 38% in the third quartile, and 52% in the fourth quartile compared to the first quartile (**Table 3**-Model III).

**Table 3:** Relationship between RC and regression to normoglycemia from prediabetes in different models.

Exposure	Model I (HR,95%CI) P	Model II (HR,95%CI) P	Model III (HR,95%CI) P	Model IV (HR,95%CI) P
RC	0.49 (0.47, 0.51) <0.0001	0.56 (0.54, 0.58) <0.0001	0.49 (0.47, 0.52) <0.0001	0.49 (0.47, 0.51) <0.0001
RC quartiles				
Q1	Ref	Ref	Ref	Ref
Q2	0.75 (0.72, 0.78) <0.0001	0.80 (0.77, 0.83) <0.0001	0.80 (0.77, 0.83) <0.0001	0.78 (0.75, 0.81) <0.0001
Q3	0.57 (0.55, 0.60) <0.0001	0.63 (0.61, 0.66) <0.0001	0.62 (0.60, 0.65) <0.0001	0.60 (0.57, 0.62) <0.0001
Q4	0.43 (0.41, 0.44) <0.0001	0.50 (0.48, 0.52) <0.0001	0.48 (0.46, 0.50) <0.0001	0.46 (0.44, 0.48) <0.0001
P for trend	<0.0001	<0.0001	<0.0001	<0.0001

Model I: we did not adjust other covariates.

Model II: we adjusted age, gender.

Model III: we adjusted age, gender, SBP, DBP, BMI, ALT, BUN, Scr, TG, family history of diabetes, drinking status, smoking status and FPG at baseline.

Model IV: we adjusted age(smooth), gender, SBP (smooth), DBP (smooth), BMI (smooth), BUN (smooth), Scr (smooth), ALT (smooth), TG (smooth), smoking status, drinking status, family history of diabetes and FPG at baseline (smooth). HR, Hazard ratios; CI, confidence; Ref, reference.

### 3.5 Sensitivity analysis

We performed a systematic sensitivity analysis to evaluate the robustness of our findings. One method involved employing a GAM to incorporate continuous covariates in a curvilinear manner into the equation. The results of this analysis concurred with those from the fully adjusted model (Model IV) (**Supplementary Table S2**). Specifically, the analysis indicated that RC was negatively associated with the likelihood of normalizing blood glucose, yielding a hazard ratio (HR) of 0.49 and a 95% confidence interval (CI) of 0.47 to 0.51. Additionally, we conducted a sensitivity analysis among participants with a BMI greater than 18 kg/m² (n=7953). After adjusting for confounders, the results showed a significant negative association between RC and the probability of normalizing blood glucose in prediabetic patients, with an HR of 0.50 and aCI of 0.48 to 0.52. This negative correlation remained significant among patients under 60 years of age (n=2232) after adjusting for confounders (HR=0.51, 95% CI 0.48–0.53). Excluding participants with a systolic blood pressure above 140 mmHg (n=1461), the Cox proportional hazards regression analysis revealed an HR of 0.48 (95% CI 0.46, 0.51) for the relationship between RC and the likelihood of normalizing blood glucose. Finally, the sensitivity analysis of the multivariate adjusted models indicated that patients in the third and fourth quartiles of RC had a significantly reduced likelihood of normalizing blood glucose compared to those in the first quartile (**Supplementary Table S2**). Our findings stood the test of all sensitivity analyses, confirming their robustness.

The results of the competing risk analysis, which considered the progression from Prediabetes to diabetes as a competing event for the reversal to normoglycemia, are presented in **Supplementary Table S3**. The results of this table indicate that there is a negative correlation between serum cholesterol levels and the reversal from prediabetes to normoglycemia. Specifically, the higher the serum cholesterol level, the lower the risk of reversal to normoglycemia. This relationship persists even after adjusting for age, gender, blood pressure, BMI, liver function, kidney function, blood lipids, family history of diabetes, alcohol consumption, smoking status, and baseline fasting plasma glucose.

### 3.6 The Cox proportional hazards regression modeling with cubic spline functions to address nonlinearity

**Figure 2:**
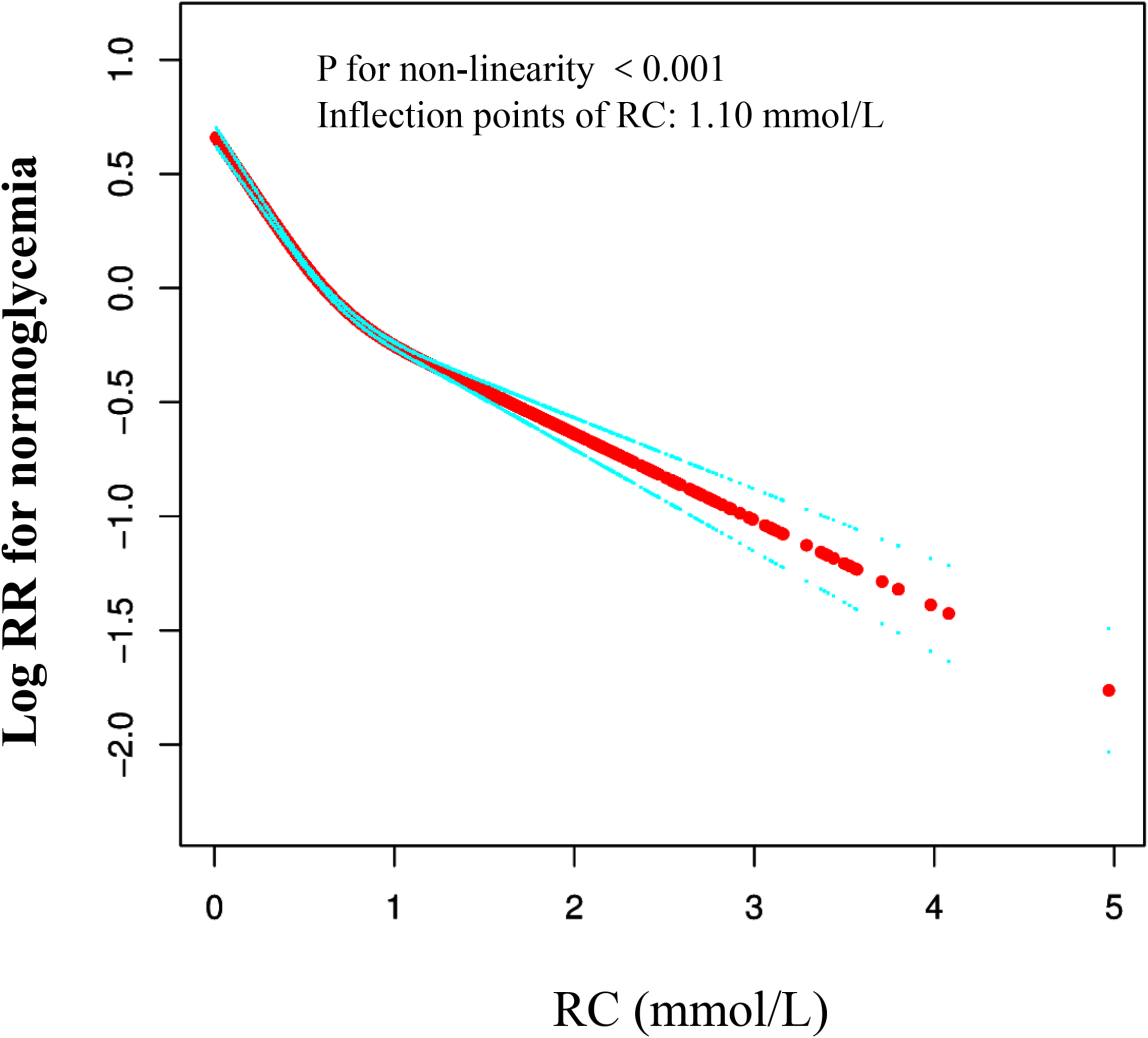
The nonlinear relationship between RC and reversion to normoglycemia from Prediabetes in Chinese non-obese adults. The result showed that the relationship between RC and regression to normoglycemia from Prediabetes was nonlinear, with the inflection point of RC being 1.47 mmol/L. (Age, gender, SBP, DBP, BMI, ALT, BUN, Scr, TG, FPG at baseline, family history of diabetes, drinking status, and smoking status were all adjusted).

We used a Cox proportional hazards regression model with cubic splines to demonstrate a non-linear association between the recovery of normal blood glucose and RC, with a correlation test yielding P < 0.001 (**Table 4**, **Figure 2**). A recursive method was used to determine the inflection point of RC, which was identified as 1.10 mmol/L. Subsequently, we computed the HRs and their CI on either side of the inflection point with a piecewise the Cox proportional hazards regression model. Beyond the inflection point, the HR was 0.83 (95% CI 0.76–0.90). Pre-inflection, the HR was 0.40 (95% CI 0.38–0.43 per unit increase), indicating a significant negative relationship in this interval.

**Table 4:** The result of the two-piecewise the Cox proportional hazards regression model.

Outcome: reversion to normoglycemia from prediabetes	HR, 95%CI	P-value
Fitting model by standard the Cox proportional hazards regression	0.49 (0.47, 0.52)	<0.0001
Fitting model by two-piecewise Cox proportional hazards regression		
Inflection points of RC (mmol/L)	1.10	
<1.47 mmol/L	0.40 (0.38, 0.43)	<0.0001
≥ 1.47 mmol/L	0.83 (0.76, 0.90)	<0.0001
P for log-likelihood ratio test		<0.001

Age, gender, SBP, DBP, BMI, ALT, BUN, Scr, TG, FPG at baseline, family history of diabetes, drinking status, and smoking status were all adjusted.

### 3.7 Subgroup analyses results

In this study, we analyzed both predefined and exploratory subgroups to gain a comprehensive understanding of the effects of various variables (as detailed in **Table 5**). The variables examined included age, BMI, SBP, DBP, gender, drinking status, and smoking status. These variables are of great importance in understanding population health status and its relationship with related diseases. However, through in-depth analysis of the data, we found that there were no significant interactions between these variables and RC, especially statistically, with all P values being greater than 0.05.

**Table 5:** Stratified associations between RC and the reversion to normoglycemia from prediabetes by age, gender, SBP, DBP, smoking status, drinking status.

Variable	Number	HR (95% CI)	P-value	P for reaction
Age(years)				0.4110
<60	5,877	0.47 (0.45, 0.50) <0.0001	<0.0001	
≥60	2,232	0.49 (0.45, 0.53) <0.0001	<0.0001	
BMI (kg/m^2^)				0.0845
<18	156	0.61 (0.48, 0.77)	<0.0001	
≥18	7,953	0.49 (0.47, 0.51)	<0.0001	
SBP (mmHg)				0.2611
≥140	1,461	0.49 (0.47, 0.51)	<0.0001	
<140	6,647	0.52 (0.47, 0.57)	<0.0001	
DBP (mmHg)				0.0633
≥90	7,322	0.49 (0.47, 0.51)	<0.0001	
<90	786	0.55 (0.49, 0.61)	<0.0001	
Gender				0.2420
Male	4,559	0.46 (0.44, 0.49)	<0.0001	
Female	3,550	0.54 (0.51, 0.57)	<0.0001	
Drinking status				0.8283
Current smoker	74	0.53 (0.41, 0.69)	<0.0001	
Ever smoker	415	0.48 (0.41, 0.55)	<0.0001	
Never	1,927	0.49 (0.45, 0.52)	<0.0001	
Unknown	5,693	0.50 (0.47, 0.52)	<0.0001	

Above model adjusted for age, gender, SBP, DBP, BMI, ALT, BUN, Scr, TG, FPG at baseline, family history of diabetes, drinking status, and smoking status. In each case, the model is not adjusted for the stratification variable when the stratification variable was a categorical variable.

## 4 Discussion

We conducted a retrospective cohort study to investigate and analyze non-obese prediabetic patients. The results reveal a significant negative correlation between RC levels and the likelihood of normal blood glucose recovery in these patients. Specifically, high RC levels substantially decrease the likelihood of patients recovering normal blood glucose. Furthermore, the study identified a critical inflection point at 1.10 mmol/L for RC, indicating that exceeding this threshold intensifies the negative impact on patients’ blood glucose recovery. This finding has significant implications for clinical practice, providing a new perspective on managing non-obese prediabetic patients and highlighting the importance of reducing residual cholesterol levels.

Several studies have demonstrated an inverse relationship between RC levels and the likelihood of blood glucose normalization in prediabetic individuals. A British cohort study found that 54% of individuals with prediabetes reverted to normoglycemia within 12 months, with only 6% progressing to diabetes mellitus (25). Similarly, Chinese cohort studies reported that about 45% of adults with prediabetes achieved normalized blood glucose levels (26). These observations highlight the pivotal role of RC levels in the reestablishment of blood glucose homeostasis. A meta-analysis has confirmed that overweight/obesity is linked to an enhanced risk of prediabetes/DM, however, further research in Asian populations is needed to substantiate the relationship between underweight and prediabetes/DM (27), Previous studies have shown that a considerable proportion (13%) of non-obese young adults suffer from prediabetes (28). Chinese researchers have initiated studies to explore the link between dyslipidemia and prediabetes in non-obese populations. This research reveals a positive, non-linear correlation between TG/HDL-C ratio and prediabetes incidence in non-obese Chinese individuals with normal LDL-C levels, highlighting the diagnostic significance of lipid parameters in this population (29, 30). The current study aims to determine the critical cutoff value of residual cholesterol for reversing prediabetes to normoglycemia in non-obese individuals, as its role in this demographic remains unclear. From a clinical treatment standpoint, the strategic reduction of residual cholesterol to beneath the identified threshold is of therapeutic importance.

This study is the first to examine the relationship between RC and the restoration of normal blood glucose levels in non-obese individuals with prediabetes, addressing a gap in current research. Sensitivity analysis revealed that the association between RC and blood glucose restoration persists across diverse subgroups, including those with BMI > 18 kg/m², systolic BP < 140 mmHg, and age < 60 years. This suggests that the relevance of RC is consistent across subgroups. Additionally, the study identifies a non-linear relationship between RC and the restoration of normal blood glucose in non-obese prediabetic individuals. Specifically, Cox proportional hazards regression model to analyze data and determined that a RC level of 1.10 mmol/L is a critical inflection point. Below the 1.10 mmol/L threshold, a one-unit reduction in RC is associated with a significant 60% increase in the probability of restoring normal blood glucose. However, when RC exceeds 1.10 mmol/L, the likelihood of restoring normal blood glucose does not significantly change. This finding provides critical guidance for clinical practice, highlighting the importance of RC in the transition from prediabetes to normal blood glucose levels in non-obese individuals. It suggests that stricter control of RC is essential in managing these patients to promote the restoration of blood glucose levels.

The inverse relationship between RC and the probability of normoglycemia reversion in prediabetes patients may be explained by various mechanisms. Firstly, RC is known to be associated with IR, which is a key characteristic of prediabetes. Abnormal blood lipid levels contribute to inflammation, endoplasmic reticulum stress, and lipid toxicity, ultimately resulting in insulin resistance (IR), which impairs glucose metabolism and elevates blood glucose levels (31–33). Therefore, higher RC levels may exacerbate insulin resistance and further impair glucose metabolism in prediabetes patients. Secondly, RC is associated with various metabolic disorders, such as dyslipidemia and hypertension, which are common in prediabetes patients (34). These metabolic disorders can further impair glucose metabolism and increase the risk of prediabetes progression to diabetes. Thirdly, elevated levels of RC may be associated with endothelial dysfunction (35, 36), which may affect blood supply to the pancreas and liver, thereby influencing glucose regulation.

Our study analyzed data from a well-defined Chinese cohort to examine the relationship between residual cholesterol and prediabetes in non-obese individuals, and to determine the optimal threshold for residual cholesterol control needed to reverse prediabetes to normoglycemia. This finding is crucial for preventing diabetes in this population. Further research is needed to clarify the mechanisms of action.

## 5 Limitation

However, we must acknowledge several limitations. First, there is a potential for sample selection bias. The study relies on a retrospective cohort design, which may introduce selection bias as participants were drawn from health check-ups, potentially excluding individuals with more severe health issues who might not seek regular check-unsecond, the focus on non-obese individuals in China limits the applicability of the results to other ethnic groups or populations with different obesity profiles. Therefore, our findings need to be validated in diverse racial populations. Third, despite adjusting for multiple covariates, unmeasured confounding factors like dietary habits, physical activity, or genetic predispositions may still affect the relationship between RC levels and reversion to normoglycemia. The 2.87-year median follow-up period may be inadequate for assessing long-term glucose metabolism outcomes and the impact of remnant cholesterol levels, given the chronic nature of prediabetes and diabetes. Fifth, the study does not provide information on any interventions or lifestyle changes that participants may have undertaken during the follow-up period, which could significantly impact the outcomes. This retrospective observational study suggests an independent association between RC and the reversion to normoglycemia in prediabetic patients, but does not establish causality. This study defined prediabetes based solely on FPG levels, without incorporating glycated hemoglobin (HbA1c) or oral glucose tolerance test (OGTT) indicators, which may have omitted some individuals with prediabetes. Future studies should further include these indicators to improve the comprehensiveness of diagnosis. Lastly, classifying participants as prediabetic based solely on fasting plasma glucose levels may overlook other important diagnostic criteria, such as HbA1c levels or oral glucose tolerance tests. Therefore, the identified prediabetic population may be underestimated. Despite the challenges of obtaining HbA1c measurements or conducting 2-hour oral glucose tolerance tests on a large cohort, future efforts should prioritize collecting 2-hour plasma glucose and HbA1c data to enhance the accuracy of cohort validation.

## 6 Conclusion

There exists a significant negative and nonlinear relationship between RC levels and the reversion to normoglycemia in non-obese Chinese prediabetic patients. These findings underscore the importance of considering RC levels in the clinical management of prediabetes and the potential for RC as a valuable biomarker for predicting disease progression.

## Data Availability

The raw data supporting the conclusions of this article will be made available by the authors, without undue reservation.
