# Supplementary material for: Association between remnant cholesterol levels and reversion to normoglycemia from prediabetes: a 5-year longitudinal cohort study of Chinese non-obese adults
Source: Front Endocrinol (Lausanne). 2025 Jul 30;16:1510470. doi: 10.3389/fendo.2025.1510470 (PMC12343219; doi:10.3389/fendo.2025.1510470)
Supplement: Supplementary file 1 [file DataSheet1.docx]

**Association between remnant cholesterol levels and reversion to normoglycemia from prediabetes: a 5-year longitudinal cohort study of Chinese non-obese adults**

Wei Liu^1†^, Wenjing Jian^2†^, Suina Lin^3†^, Zhenhua Huang^4,5*^

1 Department of Emergency Medicine, Huangpu People’s Hospital of Zhongshan, Zhongshan, 528429, China

2 Department of Integrated Traditional Chinese and Western Medicine, the First Affiliated Hospital of Shenzhen University &Shenzhen Second People’s Hospital, Shenzhen，518035, China

3 Department of Ophthalmology, Huangpu People’s Hospital of Zhongshan, Zhongshan, 528429, China

4 Department of Emergency Medicine, Pengpai Memorial Hospital, Shanwei, 516499, China

5 Department of Emergency Medicine, the First Affiliated Hospital of Shenzhen University &Shenzhen Second People’s Hospital, Shenzhen，518035, China

**Table S1** **factors of reversal to normoglycemia from prediabetes analyzed by univariate Cox proportional hazards regression.**

| Variable | Characteristics | OR (95% CI) | P-value |
| --- | --- | --- | --- |
| Age (years) | 50.29 ± 13.85 | 0.97 (0.97, 0.98) | <0.0001 |
| Gender (n, %) |  |  |  |
| Male | 4,559 (56.22%) | Ref |  |
| Female | 3,550 (43.78%) | 1.18 (1.11, 1.25) | <0.0001 |
| Hight(cm) | 165.88 ± 8.38 | 1.00 (1.00, 1.01) | 0.4990 |
| Weight(kg) | 61.90 ± 8.39 | 0.99 (0.98, 0.99) | <0.0001 |
| BMI (kg/cm^2^) | 22.41 ± 1.83 | 0.91 (0.90, 0.93) | <0.0001 |
| SBP (mmHg) | 124.47 ± 17.59 | 0.99 (0.99, 0.99) | <0.0001 |
| DBP (mmHg) | 76.17 ± 10.65 | 0.99 (0.98, 0.99) | <0.0001 |
| TC (mmol/L) | 4.99 ± 0.95 | 0.87 (0.84, 0.90) | <0.0001 |
| TG (mmol/L) | 1.53 ± 1.17 | 0.89 (0.86, 0.92) | <0.0001 |
| HDL-C (mmol/L) | 1.38 ± 0.30 | 1.87 (1.70, 2.07) | <0.0001 |
| LDL-C (mmol/L) | 2.90 ± 0.72 | 0.90 (0.86, 0.94) | <0.0001 |
| RC (mmol/L) | 0.71 ± 0.46 | 0.49 (0.45, 0.53) | <0.0001 |
| FPG at baseline (mmol/l) | 5.92 ± 0.31 | 0.22 (0.19, 0.26) | <0.0001 |
| ALT (U/L) | 18.40 (13.70-26.00) | 0.99 (0.99, 1.00) | <0.0001 |
| AST (U/L) | 22.80 (19.00-27.00) | 0.99 (0.99, 1.00) | 0.0054 |
| BUN (mmol/L) | 4.91 ± 1.24 | 0.96 (0.93, 0.98) | 0.0013 |
| Scr (mmol/L) | 70.86 ± 16.01 | 1.00 (1.00, 1.00) | 0.0175 |
| **Smoking status (n, %)** |  |  |  |
| Current smoker | 495 (6.10%) | Ref |  |
| Ever smoker | 102 (1.26%) | 1.07 (0.80, 1.44) | 0.6435 |
| Never | 1819 (22.43%) | 1.24 (1.07, 1.43) | 0.0031 |
| Unknown | 5693 (70.21%) | 1.01 (0.88, 1.15) | 0.9122 |
| **Drinking status** (n, %) |  |  |  |
| Current drinker | 74 (0.91%) | Ref |  |
| Ever drinker | 415 (5.12%) | 1.24 (0.86, 1.79) | 0.2542 |
| Never | 1927 (23.76%) | 1.16 (0.82, 1.65) | 0.4059 |
| Unknown | 5693 (70.21%) | 1.00 (0.70, 1.42) | 0.9946 |
| **Family history of diabetes** (n, %) |  |  |  |
| No | 7891 (97.31%) | Ref |  |
| Yes | 218 (2.69%) | 0.82 (0.68, 1.00) | 0.0472 |

**Table S2** **Relationship between RC and the reversal to normoglycemia from prediabetes in different sensitivity analyses.**

| Exposure | Model I (OR,95%CI) P | Model II (OR,95%CI) P | Model III (OR,95%CI) P |
| --- | --- | --- | --- |
| RC (mmol/L) | 0.50 (0.48, 0.52) <0.0001 | 0.51 (0.48, 0.53) <0.0001 | 0.48 (0.46, 0.51) <0.0001 |
| RC quartiles |  |  |  |
| Q1 | Ref | Ref | Ref |
| Q2 | 0.79 (0.76, 0.82) <0.0001 | 0.82 (0.79, 0.85) <0.0001 | 0.80 (0.77, 0.84) <0.0001 |
| Q3 | 0.61 (0.59, 0.64) <0.0001 | 0.64 (0.61, 0.67) <0.0001 | 0.62 (0.59, 0.65) <0.0001 |
| Q4 | 0.48 (0.45, 0.50) <0.0001 | 0.50 (0.47, 0.52) <0.0001 | 0.48 (0.46, 0.51) <0.0001 |
| P for trend | <0.0001 | <0.0001 | <0.0001 |

Model I involved a sensitivity analysis with participants (N=7953) whose BMI＞18 kg/m^2^. Age, gender, SBP, DBP, BMI, ALT, BUN, Scr, TG, FPG at baseline, family history of diabetes, drinking status, and smoking status were all adjusted.

Model II was a sensitivity analysis conducted on participants (N=2,232) who had age ＜60 years. Age, gender, SBP, DBP, BMI, ALT, BUN, Scr, TG, FPG at baseline, family history of diabetes, drinking status, and smoking status were all adjusted.

Model III involved a sensitivity analysis after excluding participants with SBP ＜140mmHg (N=1,461). Age, gender, SBP, DBP, BMI, ALT, BUN, Scr, TG, FPG at baseline, family history of diabetes, drinking status, and smoking status were all adjusted. CI stands for “confidence interval,” and “Ref” stands for “reference”.

**Table S3** **Relationship between RC and the reversal to normoglycemia from prediabetes in in diferent models with competing risk of progression to diabetes.**

| Exposure | Model I (SHR,95%CI) P | Model II (SHR,95%CI) P | Model III (SHR,95%CI) P |
| --- | --- | --- | --- |
| RC (mmol/L) | 0.49 (0.43, 0.52) <0.0001 | 0.50 (0.49, 0.53) <0.0001 | 0.51 (0.49, 0.53) <0.0001 |
| RC quartiles |  |  |  |
| Q1 | Ref | Ref | Ref |
| Q2 | 0.81 (0.77, 0.84) <0.0001 | 0.89 (0.82, 0.93) <0.0001 | 0.85 (0.80, 0.87) <0.0001 |
| Q3 | 0.66 (0.63, 0.70) <0.0001 | 0.68 (0.65, 0.71) <0.0001 | 0.60 (0.58, 0.64) <0.0001 |
| Q4 | 0.55 (0.52, 0.59) <0.0001 | 0.53 (0.50, 0.56) <0.0001 | 0.50 (0.47, 0.52) <0.0001 |
| P for trend | <0.0001 | <0.0001 | <0.0001 |

Model I: we did not adjust other covariates.

Model II: we adjusted age, gender.

Model III: we adjusted age, gender, SBP, DBP, BMI, ALT, BUN, Scr, TG, family history of diabetes, drinking status, smoking status and FPG at baseline.


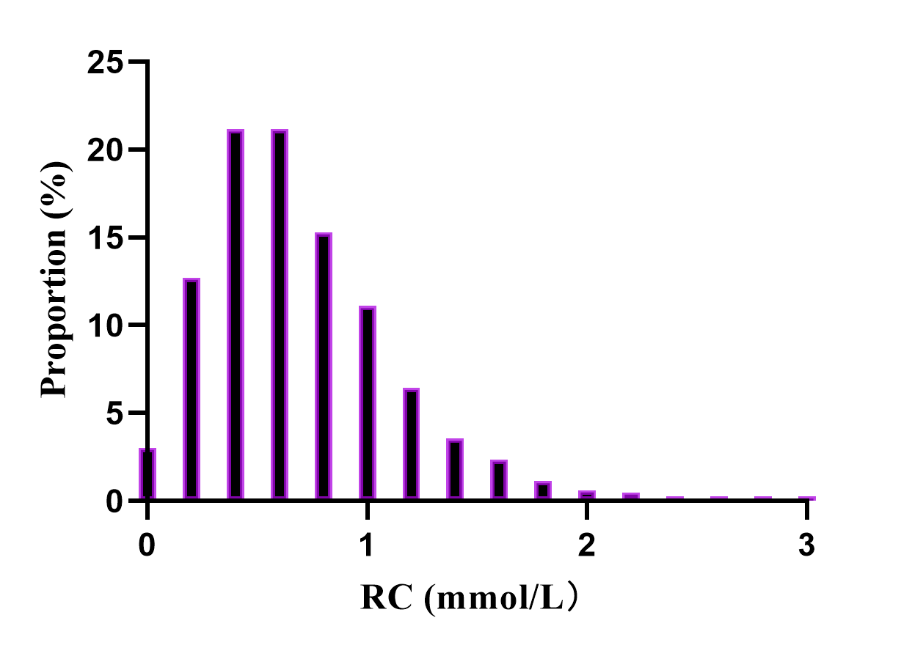


Fig S1 Distribution of RC. It presented a skewed distribution, ranging from 0.01 to 6.85 mmol/L, with a Median of 0.63 mmol/L.


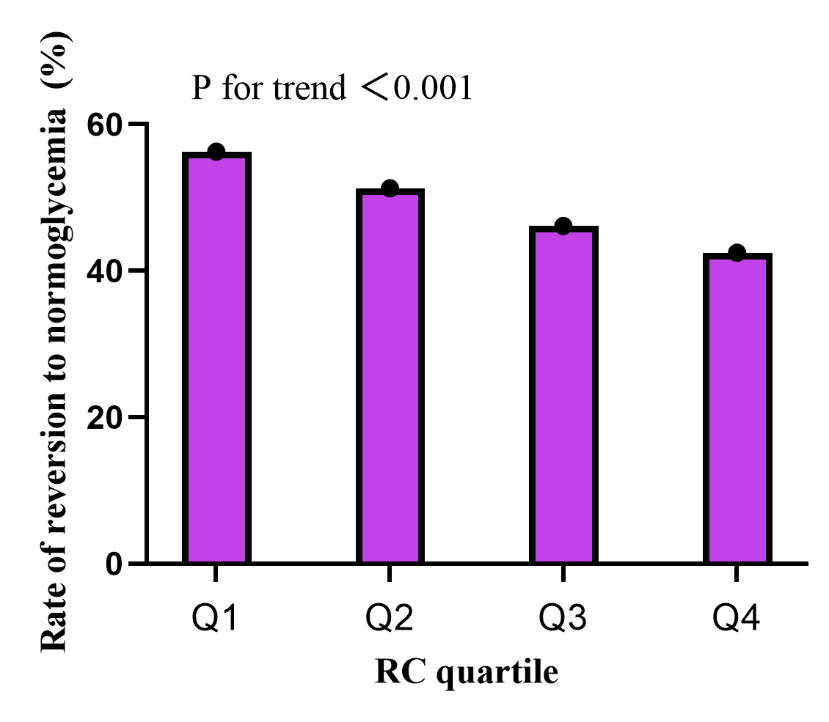


Fig S2 The rate of reversion to normoglycemia from prediabetes according to the quartiles of RC. Participants with the highest RC (Q4) had a lower regression rate to normoglycemia than those with the lowest RC (Q1) (p＜0.001 for trend).


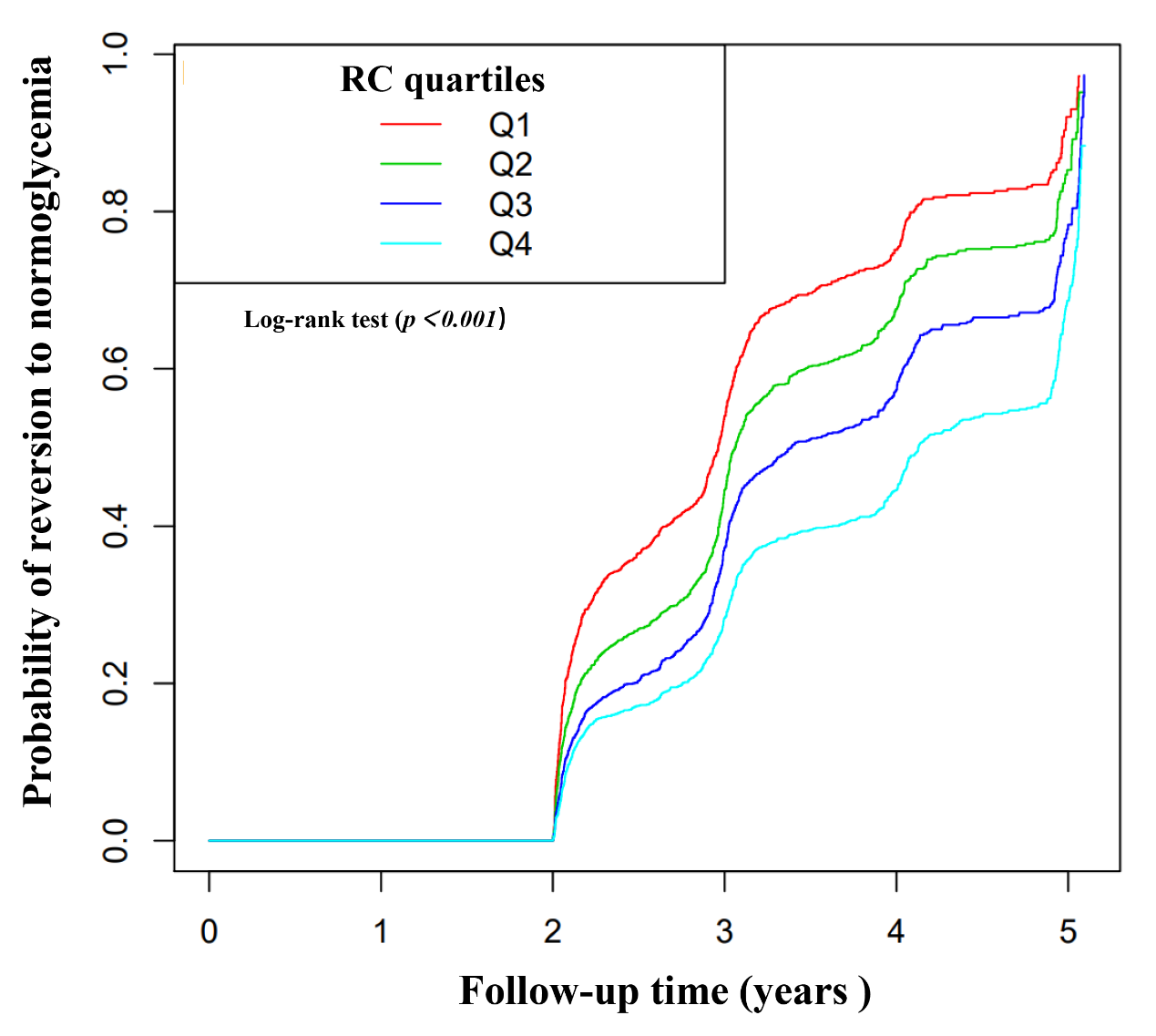


Fig S3 showed the Kaplan-Meier curves for reversion to normoglycemia form prediabetes. Displayed the Kaplan-Meier curves illustrating the probability of regression from Prediabetes to normoglycemia based on RC quartiles. There was a significant difference in the probability of returning to normoglycemia in the different RC quartile groups (log-rank test, P<0.001). Compared with participants in the lowest RC quartile group (Q1), participants in the higher RC quartile groups (Q2 to Q4) had a lower probability of reversing to normoglycemia during the follow-up period.
